# Is dietary pattern of schizophrenia patients different from healthy subjects?

**DOI:** 10.1186/1471-244X-7-15

**Published:** 2007-05-02

**Authors:** Reza Amani

**Affiliations:** 1 Dept. of Nutrition, Faculty of Paramedicine, Jundi-Shapour University of Medical Sciences, Ahvaz, Iran

## Abstract

**Background:**

There are limited findings about dietary patterns and food preferences among patients suffering from schizophrenia. The main objective of this study was therefore to compare the nutritional pattern of schizophrenia patients with that of matched healthy subjects.

**Methods:**

The dietary pattern of 30 hospitalized 16–67 years old schizophrenic patients (11 female) was compared with that of 30 healthy age and sex matched individuals as control group. Subjects' anthropometric measurements including weight, height and body mass index (BMI), semi-quantitative food frequency (FFQ), medical and food history questionnaires were also collected and FFQs were then scored using Food Guide Pyramid to obtain the dietary scores. Percent body fat (%BF) was measured using bioelectrical impedance analysis (BIA) method.

**Results:**

Female patients had more %BF and lower dietary pattern scores than that of their controls (32 ± 3.6 vs 27.7 ± 4.6 percent and 43.2 ± 11.9 vs 54.5 ± 10.7 points; respectively, p < 0.05 for both). They also consumed less milk and dairy products, fresh vegetables, fruits, chicken, and nuts compared with the female controls (p < 0.03). However, these patients used to eat more full-fat cream and carbonated drinks (p < 0.05). Male patients had lower BMI (22 ± 4.7 vs 25.6 ± 4.4; p < 0.05) than their counterpart controls but there was no significant difference between their %BFs. Moreover, they used to have more full-fat cream, hydrogenated fats, less red meat and nuts compared with the male controls (p < 0.05).

**Conclusion:**

Schizophrenia patients have poor nutritional patterns. In particular, female patients have more percent body fat and lower dietary pattern scores compared with their healthy controls. All patients used to consume more fats and sweet drinks frequently. The findings of this study suggest that schizophrenia patients need specific medical nutrition therapies through limiting dietary fats and sugars intakes and weight control. Whether obesity is the consequence of disease, dietary preference or medications used remains to be cleared.

## Background

While it is well recognized that the pattern of food intake is of substantial importance in the etiology of physical diseases such as diabetes, cancer, and cardiovascular disease and in the view of the clinical and epidemiological association between these mental and physical illnesses, it is surprising that there has been little research finding in the relationship between nutrition and mental illness [1-3]. On the other hand, some physical illnesses, particularly diabetes and coronary heart disease, occur with increased frequency in patients with schizophrenia and major depression [2,4].

Peet compared the existing database on international variations in the outcome of schizophrenia and showed that more intake of refined sugar, meat, eggs, and with a lesser degree dairy products and alcohol, was associated with a greater prevalence of depression and poor outcome of schizophrenia [1]. There was a negative (beneficial) relationship between pulses, fish and seafood intake and severity of schizophrenia (expressed as hospital admission and social outcome) and prevalence of depression, respectively [1]. Previously, Christensen and Christensen showed that there is a very strong correlation between a low percentage of total dietary fat and fat from land animals and birds (mainly saturated fats) and a good prognosis of schizophrenia. A high percentage of dietary unsaturated fatty acids was less strongly associated [5]. Peet in his review has concluded that high saturated fats, high glycemic load, and low omega-3 polyunsaturated fatty acids (PUFA) may be detrimental to the symptoms of schizophrenia [6].

However, at present, there are limited findings on nutritional status and dietary practices of schizophrenia patients. Therefore, this study has been carried out to compare the food intake of schizophrenic patients with that of healthy subjects and to find more practical nutritional recommendations for this type of patients.

## Methods

### Subjects

Thirty schizophrenia patients (11 females) within range of 16–76 years hospitalized in psychiatry ward were recruited. They were diagnosed and admitted by the attending psychiatrists. Patients had more than one-year history of disease and hospitalized in Ahvaz University Golestan Medical Center at least for one week. Controls (n = 30; 14 female) were age-matched (within 5 years) individuals selected from the patients' siblings, friends and/or people who have been living within similar areas compared to the patients and had no specific history of psychiatric, psychologic, neurologic, and metabolic diseases. Moreover, there was no statistically significant difference in sex distribution between groups. All patients' families declared their consents verbally and they were assured that all information will be confidential and no blood sampling will be done in this study. Moreover, there was no need to reveal the patients' names. Medical Ethics Committee of Jundi-Shapour University has approved the study protocol.

### Measurements

Data collection was carried out during spring 2004. Variables collected were anthropometric indices including weight and height, socio-economic status, medical and food histories. Semi-quantitative food frequency questionnaires were also completed. In these questionnaires, the patients' usual dietary patterns were recorded. Subject's weight and height were measured using Seca digital scale; Germany, and a non-stretchable wall meter, respectively. Individual's body mass index (BMI) was calculated as weight (Kg) divided by squared height (m^2^) and subject's percent body fat (%BF) was measured using the bioelectrical impedance analysis (BIA) method by OMRON BF-302; Japan with standard errors of estimate (accuracy) of 4.1%. Food frequency questionnaires were then scored on the basis of the Food Guide Pyramid and Mediterranean Pyramid and interpreted as dietary scores.

### Statistics

Data were analyzed by independent-t and Chi-square tests using SPSS soft-ware version # 11.5. P value less than 0.05 was regarded as significant.

## Results

There was no significant difference between the mean age of the patients and the controls. Table 1 represents the anthropometric criteria and dietary scores of both female groups. Dietary scores of female patients were significantly lower while their %BFs were higher than that of their controls (p < 0.05). Male patients were shorter while their BMIs were higher than their controls (p < 0.05).

**Table 1:** Basic characteristics of the subjects†

**Criteria**	**Male**		**Female**	
	**Patients**	**Controls**	**Patients**	**Controls**
**Age (y)**	32. 3 ± 9	35.6 ± 14	32.5 ± 9.2	36.6 ± 9.5
**Weight (Kg)**	68.4 ± 14	73.7 ± 10.6	66.9 ± 10.7	66.8 ± 12.3
**Height (Cm)**	176.5 ± 7.6	170 ± 8 *	160 ± 6.3	162 ± 4.9
**BMI (Kg/m**^2^**)**	22 ± 4.7	25.6 ± 4.4*	26 ± 3.6	25.4 ± 3.8
**Percent body fat**	14.3 ± 9	14.6 ± 3	32 ± 3.6	27.7 ± 4.6*
**Dietary scores**	54.8 ± 6.7	57 ± 7.7	43.2 ± 11. 9	54.5 ± 10.7*

† Data are shown as: Mean ± SD.

* p < 0.05

Table 2 shows the consumption of the main food groups in study groups based on their sexes. More male patients used to eat hydrogenated fats (about 2.3 times more; p = 0.03), and full-fat cream (7 times more; p = 0.005) but fewer ate red meats (about one eighth; p = 0.02), vegetable oils (p = 0.001), and nuts (p = 0.01) servings per day compared with their controls. More female patients used to drink carbonated drinks (p = 0.006) but fewer consumed milk, vegetables, nuts, and sausages servings daily (P < 0.05 for all).

**Table 2:** Consumption of food groups in schizophrenia patients and their controls separated by sex.^1^

		**Male(n = 35)**					**Female(n = 25)**				
**Food groups**	Servings*	**Patients**		**Controls**		**p value**	**Patients**		**Controls**		**p value**
		No	%	No	%		No	%	No	%	

**Milk**	1–2/d	6	32	5	31	0.9	2	18	5	36	0.03
**Vegetables**	1–2/d	13	68	7	44	0.6	4	36	6	43	0.04
**Fruits**	3/d	10	53	12	75	0.3	7	64	14	100	0.1
**Meats**	1–2/d	1	5	6	38	0.02	2	18	7	50	0.08
**Tuna fish**	1–2/wk	5	26	7	44	0.2	3	27	4	29	0.9
**Chicken**	1–2/d	15	79	16	100	0.2	5	45	14	100	0.02
**Eggs**	1–2/d	15	79	12	75	0.3	5	45	10	71	0.3
**Hydrogenated**											
**Oils**	3–5/d	16	83	13	37	0.03	7	64	11	79	0.3
**Vegetables**											
**Oils**	3–5/d	10	53	15	94	0.001	5	45	11	79	0.08
**Cream**	1–2/d	8	42	1	6	0.005	2	18	0	0	0.2
**Nuts**	1–2/d	2	10	4	25	0.01	2	18	10	71	0.03
**Carbonated**											
**Drinks**	1–2/d	4	21	3	19	0.08	4	36	0	0	0.006
**Sausages**	1–2/d	10	53	4	25	0.08	2	18	5	36	0.03
**Chocolate**	1–2/d	8	42	8	50	0.08	2	18	5	36	0.2

1- Data based on semi-quantitative food frequency questionnaires.

* Servings are presented based on Food Guide Pyramid and Mediterranean Pyramid

In comparison with the female patients, more men used to eat vegetables, eggs, cream and chocolate (p < 0.05 for all) but fewer ate tuna fish servings (p = 0.02; table 3). All patients used to consume full-fat cream and chocolate in their daily food patterns.

**Table 3:** Consumption of food groups in schizophrenia patients and their controls separated by the study group.^1^

		**Patients(n = 30)**					**Controls(n = 30)**				
**Food groups**	Servings*	**Male**		**Female**		**p value**	**Male**		**Female**		**p value**
		No	%	No	%		No	%	No	%	

**Milk**	1–2/d	6	32	2	18	0.07	5	31	5	36	0.08
**Vegetables**	1–2/d	13	69	4	36	0.01	7	44	6	43	0.7
**Fruits**	1–2/d	10	53	7	64	0.2	12	75	14	100	0.07
**Meats**	1–2/d	1	5	2	18	0.6	6	38	7	50	0.5
**Tuna fish**	1–2/wk	3	16	7	64	0.02	5	31	8	57	0.3
**Chicken**	1–2/wk	15	79	5	45	0.1	16	100	14	100	-
**Eggs**	1–2/d	15	79	5	45	0.01	12	75	10	71	0.2
**Hydrogenated**											
**Oils**	3–5/d	16	84	13	81	0.02	7	64	11	79	0.3
**Vegetables**											
**Oils**	3–5/d	10	53	5	45	0.4	15	94	11	79	0.2
**Cream**	1–2/d	8	42	2	18	0.6	1	6	0	0	0.05
**Nuts**	1–2/d	2	10	2	18	0.6	4	25	10	71	0.02
**Carbonated**											
**Drinks**	1–2/d	4	21	4	36	0.3	3	19	0	0	0.01
**Sausages**	1–2/d	10	53	2	18	0.1	4	25	5	36	0.3
**Chocolate**	1–2/d	8	42	2	18	0.05	8	50	5	36	0.03

1- Data based on semi-quantitative food frequency questionnaires.

* Servings are prensented based on Food Guide Pyramid and Mediterranean Pyramid.

## Discussion

Previous studies have shown that dietary fats are associated with adverse outcome in schizophrenia [5-7]. However, diet is much more complex than this and dietary intake of different food stuff is highly intercorrelated. For example, people who eat a lot of saturated fat will generally also eat a lot of sugar [8]. It was also shown that meat, dairy products, and saturated fat intake were associated with poor outcome of schizophrenia [1,4,5].

The results of the present study showed that schizophrenia patients, especially women, had low overall dietary scores than their healthy matched controls, based on the Food Guide Pyramid [9]. Moreover, females were clinically overfat (%BF 32 ± 3.6) in comparison with their controls.

At the same time, male patients had higher BMI values than their controls (the former defined as overweight). Strassing et al have indicated that excess body weight in schizophrenic patients is associated with serious health risks. They also reported that female patients were more likely to approach weight loss than males [10]. Weiser et al followed the hospitalization of the schizophrenia patients among male adolescents who had been assessed by a national psychiatric registry for 2–6 years and they showed that these patients before the onset of illness, were not heavier than their peers and the increased weight of patients was related to illness effects e.g. antipsychotic medication [11].

In this study, majority of the male patients (83%) used to eat hydrogenated fats as well as full-fat cream daily but vegetable oils intake was much lower in comparison with their controls. It should be noted that most Iranians are traditionally consuming hydrogenated fats (ghee) in their daily diets and this is the main reason for high proportions seen even in the healthy subjects here.

Based on the results obtained female patients' overall food pattern scores were significantly lower than their controls and also the male patients.

Moreover, patients drank more carbonated drinks but fewer ate vegetable oils and nuts, both are recognized as good sources of antioxidants such as vitamin E and carotenoids and as well as essential fatty acids. By obtaining 24-h diet recall questionnaires of 146 patients with schizophrenia, Strassing et al. found that saturated and poly unsaturated fatty acid intake was significantly higher in patients than in controls, but there were no differences with regard to dietary intake of omega-3 fatty acids and antioxidants [12]. WHO have reported that countries with a higher saturated fat intake from land animal and bird sources had worse outcome of schizophrenia than countries with relatively higher PUFA intake from fish, seafood and vegetables, thereby confirming the observation of a better outcome of schizophrenia in developing countries than in industrialized countries [13]. More recently, abnormalities in cell membrane phospholipid metabolism of schizophrenia patients, including low PUFA brain content [14,15] or RBC membranes [16] have been described.

However, it is known that patients with schizophrenia make poor dietary choices [17] and their tendency to have increased caloric intake including a higher total dietary fat consumption than healthy individuals has been indicated previously [18]. Hence, becoming overfat and/or obese could enhance their risk of other complications. These findings are in agreement with those presented here. There are reports indicating that schizophrenia patients may be exposed to increased oxidative stress when compared to the population [19] which could be due to increased peroxidative stress during acute phase of disease [20], increased smoking [21] or prolonged treatment with antipsychotic medication [22].

The results presented here also showed that fewer female patients used to consume fresh vegetables than their controls and male patients. There was no difference in fruit consumption between two study groups. Both fresh fruits and vegetables are good sources of dietary antioxidants. However, antioxidants intake and their serum levels in this type of patients need further investigations.

It is worthy to note that some findings presented here, in fact, are statistical associations rather than cause and effect relationships hence, expose time and outcomes should be evaluated in more details in another setting.

Finally, this research was a pilot study and all of the patients in the ward were selected in a simple sampling procedure, therefore, case-control studies with more number of subjects, and preferably, longitudinal cohort studies, are needed to compare both the intakes and serum levels of certain nutrients. Furthermore, no clear conclusion can be made regarding cause and effect relationships between dietary practices and etiology and/or severity of schizophrenia. Because of limited information available about dietary practices and nutritional requirements in schizophrenia, the present findings can be regarded as a small step toward practical implications for clinicians and dieticians. It is suggested that all schizophrenic patients need more dietary assessment and specific nutritional cares.

## Competing interests

The author(s) declare that they have no competing interests.

## Pre-publication history

The pre-publication history for this paper can be accessed here:



## References

[B1] Peet M (2004). International variations in the outcome of schizophrenia and the prevalence of depression in relation to national dietary practices: an ecological analysis. Br J Psychiatry.

[B2] Ryan MC, Thakore JH (2002). Physical consequences of schizophrenia and its treatment: the metabolic syndrome. Life Sci.

[B3] Tucker KL, Buranapin S (2001). Nutrition and aging in developing countries. J Nutr.

[B4] Peet M, Edwards RhW (1997). Lipid, depression and physical diseases. Current Opin psychiatry.

[B5] Christensen O, Christensen E (1988). Fat consumption and schizophrenia. Acta Psychiatrica Scandinavica.

[B6] Peet M (2004). Nutrition and schizophrenia: beyond omega – 3 fatty acids. Prostaglandins, Leukotrienes and Essential Fatty Acids.

[B7] McIntosh A, Lawrie S (2004). Cross national differences in diet, the outcome of schizophrenia and the prevalence of depression: you are (associated with) what you eat. Br J Psychiatry.

[B8] Peet M (2003). Dietary predictors of schizophrenia and depression. Presented at Ninth European Nutritional Conference, Rome.

[B9] US Department of Agriculture Food and NutritionInformation Center. Food Guide Pyramid. http://www.nal.usda.gov/fnic/Fpyr/pyramid.html.

[B10] Strassing M, Brar JS, Ganguli R (2005). Self-reported body weight perception and dieting practices in community – dwelling patients with schizophrenia. Schizophr Res.

[B11] Weiser M, Knobler H, Lubin G, Nahon D, Kravitz E, Kaspi A, Noy S, Knobler HY, Davidson M (2004). Body mass index and future schizophrenia in Israeli male adolescents. J Clin Psychiatry.

[B12] Strassing M, Brar JS, Gangali R (2005). Dietary Fatty acid and antioxidant intake in community – dwelling patients suffering from schizophrenia. Schizophr Res.

[B13] WHO (1979). Schizophrenia: An International Follow – up Study.

[B14] Yao JK, Reddy RD (2002). Membrane pathology in schizophrenia: implication for arachidonic acid signaling. Scientific World Journal.

[B15] Yao JK, Leonard S, Reddy RD (2000). Membrane phospholipid abnormalities in postmortem brains from schizophrenic patients. Schizophr Res.

[B16] Assies A, Lieverse R, Vreken P, Wanders RJ, Dingemans PM, Linszen DH (2001). Significantly reduced docosahexaenoic and docosapentaenoic acid concentrations in erythrocyte membranes from schizophrenic patients compared with a carefully matched control group. Biol Psychiatry.

[B17] Browns, Birthwistle J, Roe L, Thompson C (1999). The unhealthy lifestyle of people with schizophrenia. Psychol Med.

[B18] Strassing M, Brar JS, Ganguli R (2003). National assessment of patients with schizophrenia: a preliminary study. Schizophr Bull.

[B19] Prabakaran S, Swatton JE, Ryan MM, Huffaker SJ, Huang JT, Griffin JL, Wayland M, Freeman T, Dudbridge F, Lilley KS, Karp NA, Hester S, Tkachev D, Mimmack ML, Yolken RH, Webster MJ, Torrey EF, Bahn S (2004). Mitochondrial dysfunction in schizophrenia: evidence for compromised brain metabolism and oxidative stress. Mol Psychiatry.

[B20] Reddy R, Keshavan M, Yao JK (2003). Reduced plasma antioxidants in first episode Patients with schizophrenia. Schizopr Res.

[B21] McCreadi R, On behalf of the Scotish schizophrenia Research Group (2000). Smoking habits and plasma lipid peroxide and vitamin E levels in never treated first – episode patients with schizophrenia. Br J Psychiatry.

[B22] Lohr JB, Kuczenski R, Niculescu AB (2003). Oxidative mechanisms and tardive dyskinesia. CNS Drugs.

